# Radiological Pitfalls in Patients with Inducible Dynamic Proptosis

**DOI:** 10.2174/1874364100802010091

**Published:** 2008-05-05

**Authors:** Sharon R Morris, Jean-Louis DeSousa, Ian Francis, Lekha Chandrasekharan, Raman Malhotra

**Affiliations:** 1 Corneoplastic Unit, Queen Victoria Hospital, Holtye Road, East Grinstead, West Sussex RH19 3DZ, UK; 2 Department of Radiology, Queen Victoria Hospital, Holtye Road, East Grinstead, West Sussex RH19 3DZ, UK

**Keywords:** CT, MRI, dynamic, proptosis, valsalva.

## Abstract

We report two patients presenting with marked clinical unilateral enophthalmos who had positional variability and dynamic proptosis on valsalva. On orbital imaging, enophthalmos was not documented and in fact, globe proptosis of the same side was reported for one of the patients. During CT and MRI scanning patients are often instructed to hold their breath to eliminate motion artefact. This may inadvertently induce dynamic proptosis. The radiological pitfalls of imaging patients with inducible dynamic proptosis and how to identify such patients are discussed.

## INTRODUCTION

Dynamic globe proptosis may be present spontaneously, for example, due to carotid-cavernous fistulae (classic “pulsatile proptosis”). Alternatively, it is often only inducible - the eye may only be seen to proptose forward with positional change or the valsalva manoeuvre, which is often required during routine orbital imaging. Awareness of this condition is essential in aiding the correct diagnosis and appropriate management. We report two cases that presented for surgical correction of their enophthalmos which was not detected radiologically, with a report of globe proptosis in one instance. These cases serve as a reminder of the radiological difficulties in imaging patients with inducible dynamic proptosis and how best to identify these patients.

## PATIENTS

### Case 1:

A 29 year old man had been diagnosed with a left facial and orbital low-flow arteriovenous malformation. In the past, he had undergone embolization treatment in 1983, and endoscopic ND Yag laser of the oral cavity with transcutaneous laser of the lower eyelid, cheek and upper lip in 2001. The latter was repeated in 2003.

He was referred for consideration of treatment to correct progressive enophthalmos. He was found to have 1mm medial and 2mm inferior displacement of the left globe with 7mm left enophthalmos on sitting and resting supine. On performing the valsalva manoeuvre his left enophthalmos reversed and his globe was proptosed. His optic nerve function was intact with full extraocular movements, normal intraocular pressures and fundi.

A CT scan of the orbits was performed during which the patient lay supine and was given standard departmental instructions to hold his breath, look straight ahead and remain still, in order to avoid movement artefact. The coronal images revealed demineralisation and deficiency of the orbital floor with a soft tissue mass involving the lateral orbit, face and extension into the infratemporal and pterygoid fossa and parapharyngeal space. Despite having 7mm of left enophthalmos on lying supine clinically, only minimal enophthalmos of the left globe was evident radiologically (Fig. **1a**-**c**).

Fig. (**1a**) shows a very marked left sided enophthalmos on lying supine which measured 7mm on Hertel exophthalmometry and is demonstrated in the photograph by the distance of the left globe from a horizontal line drawn at the level of the superior limbus of the other eye. On undergoing valsalva manoeuvre, this enophthalmos virtually disappears (Fig. **1b**).

The disparity between the globe position on CT and the last clinic visit necessitated a repeat assessment. This was essential to exclude a new change prior to embarking on surgical implantation of a high density porous polyethelyene (Medpor®) enophthalmic wedge implant, in order to augment his orbital volume and increase the anterior projection of his globe into a more aesthetic position.

### Case 2:

A 24 year old woman was originally referred for consideration to improve facial asymmetry due to progressive right globe enophthalmos with inferior displacement (Fig. **2a**). A diagnosis of neurofibromatosis was made and confirmed as type 1 with spontaneous mutation on the basis of multiple café au lait patches, axillary freckling, dermal neurofibromas and right sphenoid wing hypoplasia.

On examination she had 7mm medial and 3mm inferior globe displacement with marked right enophthalmos. She had no diplopia in primary position but she had a global reduction in right eye movements especially in abduction and elevation. Her optic nerve function was normal and her remaining ocular examination was unremarkable.

On lying supine, the right globe became proptosed by 1mm relative to the left eye (Fig. **2b**).

A pre-operative MRI scan was perfomed to enable surgical planning for correction of the enophthalmos. This was perfomed supine, eyes in primary position and the patient was asked to breath-hold in order to minimise movement artefact for image capture. The axial scans showed deficient greater and lesser wings of sphenoid on the right side with deficiency of the left side of the planum sphenoidale (involving the left sphenoidal sinus) and right anterior clinoid processes. Brain herniation through the bony deficiency was evident and the right globe was reported as proptosed (Fig. **2c**).

After consideration, the patient chose not to undergo surgical intervention.

Fig. (**2a**) shows marked right sided enophthalmos in the sitting position, as demonstrated by the right globe distance from a horizontal line drawn from the left cornea, with reversal and mild proptosis on lying supine (Fig. **2b**).

## DISCUSSION

Dynamic proptosis is defined as exophthalmos occurring when a variable force is applied to the orbital contents [1]. Reported aetiologies include orbital vascular malformations – such as orbital varices, carotid-cavernous fistulae, intracavernous aneurysms and combined venous lymphatic malformations (or so-called lymphangiomas), as well as bony wall abnormalities arising from neurofibromatosis, previous surgery, paranasal mucoceles and meningoencephaloceles, malignancy and dermoid cysts [1]. Dynamic proptosis may be *spontaneous* (“pulsatile proptosis”) or* inducible*, requiring positional change or the valsalva manoeuvre to elicit. Such manoeuvres are often required during routine orbital imaging. Therefore, inducible dynamic proptosis may mask marked clinical enophthalmos when imaging, as in case 1, or be misinterpreted to be static proptosis only, as in our second case.

Dynamic proptosis occurs when a variable force is applied to the orbital contents, either from haemodynamic changes to the arterial or venous system, or from absent orbital bony architecture (as above), thereby allowing transmission of brain pulsations or mechanical pressure from muscles of mastication. Therefore, manoeuvres such as forced expiration, coughing, straining, leaning forward or change in head position, jugular compression, clenching of the jaw and the valsalva manoeuvre may accentuate proptosis. During CT/MR imaging, the patient is placed supine and may be asked to breath-hold to reduce motion artefact. As a result, dynamic proptosis may be inadvertently induced.

Enophthalmos is a recognized feature in patients with orbital varices and is thought to be due to fat atrophy [2]. Orbital venous malformations may be missed on plain axial CT scans, even with contrast enhancement, but can be clearly defined with the use of the valsalva manoeuvre [3-5]. It is also well known that the dependent head position with hyperextension of the neck required for direct coronal CT scans, can better demonstrate such malformations [6]. MRI does not require changes in head position to achieve coronal images but image capture takes longer than CT and utilisation of breath-holding to avoid motion artefact is necessary. Prone positioning with increased intra-abdominal pressure has been used in MRI to specifically highlight orbital venous malformations [7].

Typically, in enophthalmic patients, imaging is used preoperatively to aid assessment of orbital volume and space for potential placement of implants. Surgical implants are used for cases where the clinical enophthalmos usually measures greater than 2mm. In the surgical planning of case 1, the size of orbital implant was based on a clinical volume assessment of the left orbit alone. Quantitative orbital volume assessment from axial CT images has been used as a predictive marker for surgical outcomes in determination of the size of orbital implant used [8]. To the best of our knowledge, quantitative assessment of dynamic orbital volume in patients with inducible proptosis has not been performed.

Currently, the speed of multiplanar image capture for CT is faster than MRI and can be performed without breath holding, although, unless specifically excluded, it remains present within most departmental protocols. Our experience would advise that when imaging is performed in cases of possible proptosis, the supine patient should be instructed to look straight ahead and breathe normally, to enable assessment of the globe position without inducing valsalva. The ophthalmologist needs to bear in mind the positioning of these patients when requesting imaging and assessing the radiological orbital volume. An alternative approach would be to use cone-beam CT. This technique is able to provide images up to the skull base with the patient in an upright position, and so allows orbital imaging without the induction of positional globe proptosis. However, access to this facility is limited.

## CONCLUSION

In summary, the above two cases highlight the radiological pitfalls in imaging enophthalmic patients with inducible dynamic proptosis. If the dynamic proptosis phenomenon is not recognized then it can lead to erroneous radiological reporting with no bearing at all on the appearance of the patient in a chair, facing the clinician. If clinical suspicion of inducible dynamic proptosis exists, then alerting the radiologist to perform positional orbital imaging with and without valsalva manoeuvre is recommended.

## Figures and Tables

**Fig. (1a):**
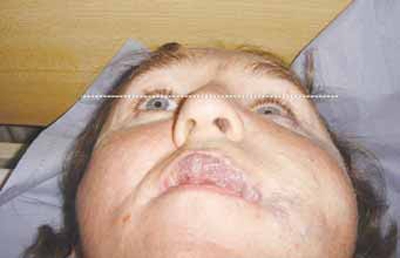
Patient lying supine, resting.

**Fig. (1b):**
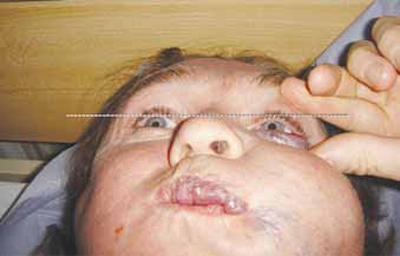
Patient supine, during valsalva.

**Fig. (1c):**
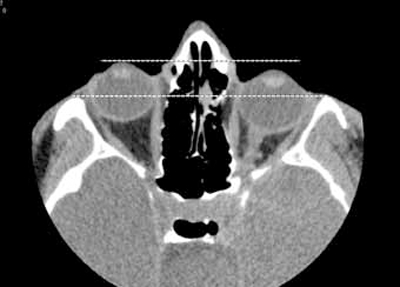
In contrast to the clinical picture, this Axial CT scan image, performed during an inadvertent Valsalva manoeuvre, shows only minimal left enophthalmos (highlighted by the broken lines).

**Fig. (2a):**
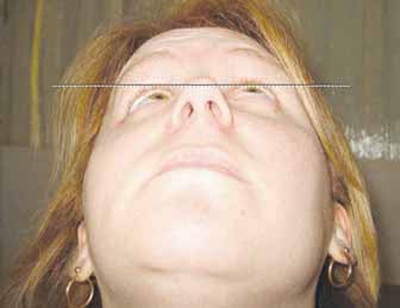
Patient sitting

**Fig. (2b):**
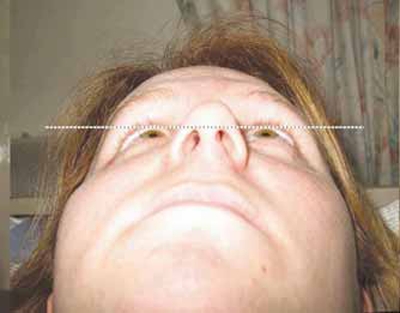
Patient lying supine, resting.

**Fig. (2c):**
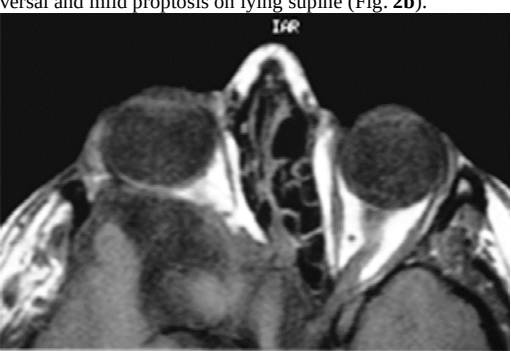
T1 weighted MRI scan showing right sided herniation of brain contents through the orbital bony structural abnormality with proptosis of the globe.

## References

[1] Bullock J, Bartley G (1986). Dynamic proptosis. Am J Ophth.

[2] Rootman J, Chang W, Jones D, Rootman J (2003). Diseases of the orbit. A multidisciplinary approach.

[3] Shields JA, Dolinskas C, Augsburger JJ (1997). Demonstration of orbital varix with computed tomography and valsalva maneuver. Am J Ophth.

[4] Takechi A, Uozumi KK, Yano T (1994). Embolization of orbital varix. Neuroradiol.

[5] Gorospe L, Royo A, Berrocal T (2003). Imaging of orbital disorders in paediatric patients. Eur Radiol.

[6] Winter J, Centeno RS, Bentson JR (1982). Maneuver to aid diagnosis of orbital varix by computed tomography. Am J Neuroradiol.

[7] Manfre L, Lagalla R, Pappalardo S (1995). Orbital varice: a tricky diagnosis in MRI. Eur Radiol.

[8] Fan X, Li J, Zhu J (2003). Computer-assisted orbital volume measurement in the surgical correction of late enophthalmos caused by blowout fractures. Ophth Plast Reconst Surg.

